# Temperature-specific adaptations and genetic requirements in a biofilm formed by *Pseudomonas aeruginosa*

**DOI:** 10.3389/fmicb.2022.1032520

**Published:** 2023-01-06

**Authors:** Karishma Bisht, Alex R. Luecke, Catherine A. Wakeman

**Affiliations:** Department of Biological Sciences, Texas Tech University, Lubbock, TX, United States

**Keywords:** biofilm, thermal adaptation, *Pseudomonas aeruginosa*, environment biofilm, host biofilm, EPS matrix

## Abstract

*Pseudomonas aeruginosa* is a gram-negative opportunistic pathogen often associated with nosocomial infections that are made more severe by this bacterium’s ability to form robust biofilms. A biofilm is a microbial community encompassing cells embedded within an extracellular polymeric substrate (EPS) matrix that is typically secreted by the encased microbial cells. Biofilm formation is influenced by several environmental cues, and temperature fluctuations are likely to be an important stimulus in the lifecycle of *P. aeruginosa* as it transitions between life in aquatic or soil environments to sites of infection in the human host. Previous work has demonstrated that human body temperature can induce a shift in the biofilm EPS relative to room temperature growth, resulting in an incorporation of a filamentous phage coat protein into the biofilm EPS. In this study, we sought to identify adaptations enabling biofilm formation at room temperature or temperatures mimicking the natural environment of *P. aeruginosa* (23°C and 30°C) relative to temperatures mimicking life in the human host (37°C and 40°C). We identified higher biofilm: biomass ratios at lower temperatures on certain substrates, which correlated with a higher relative abundance of apparent polysaccharide EPS content. However, the known genes for EPS polysaccharide production in *P. aeruginosa* PA14 did not appear to be specifically important for temperature-dependent biofilm adaptation, with the *pelB* gene appearing to be generally important and the *algD* gene being generally expendable in all conditions tested. Instead, we were able to identify two previously uncharacterized hypothetical proteins (*PA14_50070* and *PA14_67550*) specifically required for biofilm formation at 23°C and/or 30°C relative to temperatures associated with the human host. These unstudied contributors to biofilm integrity may have been previously overlooked since most *P. aeruginosa* biofilm studies tend to use 37°C growth temperatures. Overall, our study demonstrates that temperature shifts can have dramatic impacts on biofilm structure and highlights the importance of studying environment-specific adaptations in biofilm physiology.

## Introduction

Many bacterial species can form biofilms, resulting in a group of bacteria attached to a surface and surrounded by an extracellular polymeric substance (EPS) matrix which is composed of a mixture of macromolecules such as polysaccharides, proteins, carbohydrates and extracellular DNA (Donlan, 2002; Bjarnsholt, 2013). A biofilm often has a complex structure composed of differentiated groups of cells and a heightened antibiotic resistance (Donlan, 2001; Hall-Stoodley et al., 2004). *Pseudomonas aeruginosa,* a common nosocomial pathogen, forms a biofilm in the lungs of cystic fibrosis patients, in burn wounds, and on indwelling medical devices, causing infections that are largely resistant to antibiotics and to human immune defenses (Mulcahy et al., 2014; Maurice et al., 2018; Bisht et al., 2020). The EPS matrix is an important component of the biofilm since it not only provides structural stability to the biofilm but also offers functional benefits that can lead to both enhanced virulence and antimicrobial tolerance (Flemming et al., 2007; Dragoš and Kovács, 2017; Karygianni et al., 2020). Additionally, the EPS matrix is known to protect the bacterial cells from environmental stress factors like pH and osmotic stress (Hwang et al., 2016; Yan et al., 2017).

Another environmental factor that can influence a pathogen’s survival requirements is temperature. Since *P. aeruginosa* is a ubiquitous microbe that can not only infect immunocompromised humans, but can also survive in the soil, in plants, and in within streams, where temperature is different from that of a human host (Schmidt et al., 1996; Hardalo and Edberg, 1997; Mena and Gerba, 2009; Silby et al., 2011; Balcázar et al., 2015; Radó et al., 2017), studying the effect of temperature on the physiology of this important pathogen is crucial. Additionally, this pathogen can form biofilms on different surface materials ranging from medical equipment to industrial pipes, in both hospital as well as industrial settings (de Abreu et al., 2014; Bédard et al., 2016). The impact of temperature on the biofilm formation has been studied in several microbes including *P. aeruginosa* (Townsley and Yildiz, 2015; Townsley et al., 2016; Plumley et al., 2017; Kim et al., 2020; Almblad et al., 2021; del Peso Santos et al., 2021). Temperature is also known to cause stress in mesophilic pathogens found in cold water (Yi et al., 2022). Previous research has shown that temperature can indeed influence the gene expression and protein profile of *P. aeruginosa* at environmental versus host-associated temperatures (Bisht et al., 2021). The temperature fluctuations can also result in physiological adaptation and structural changes in the biofilm, which eventually help *P. aeruginosa* adapt better during its transition from the outside environment into a host.

Our goal in this study was to elucidate the effect of temperature on the EPS matrix of the biofilm formed by *P. aeruginosa* PA14 at 23°C, 30°C, 37°C and 40°C. The strain was selected as it has fewer potentially partially redundant mechanisms to promote EPS formation which could help uncover as yet unexplored biofilm EPS adaptations. Additionally, since light/dark cycling has been shown to impact PA14 biofilm gene expression, affecting both metabolic pathways and redox homeostasis, all biofilms were kept in dark for our experimental purposes (Kahl Lisa et al., 2022). The temperature range in our study was chosen to simulate conditions relevant to both industrial/environmental and medical settings, ranging from the standard room temperature of a hospital setting to temperatures associated with human fever. We identified EPS adaptations of *Pseudomonas aeruginosa* and specific genetic requirements that support biofilm formation at temperatures lower than the human host. These adaptations can serve as potential targets to further differentiate treatment methods in clinical and environmental settings, which could assist in limiting the emergence of resistance in environment reservoirs to the same antibiotics that are used in humans.

## Results and discussion

### Environmental temperature biofilm is associated with higher biomass

**Figure 1:**
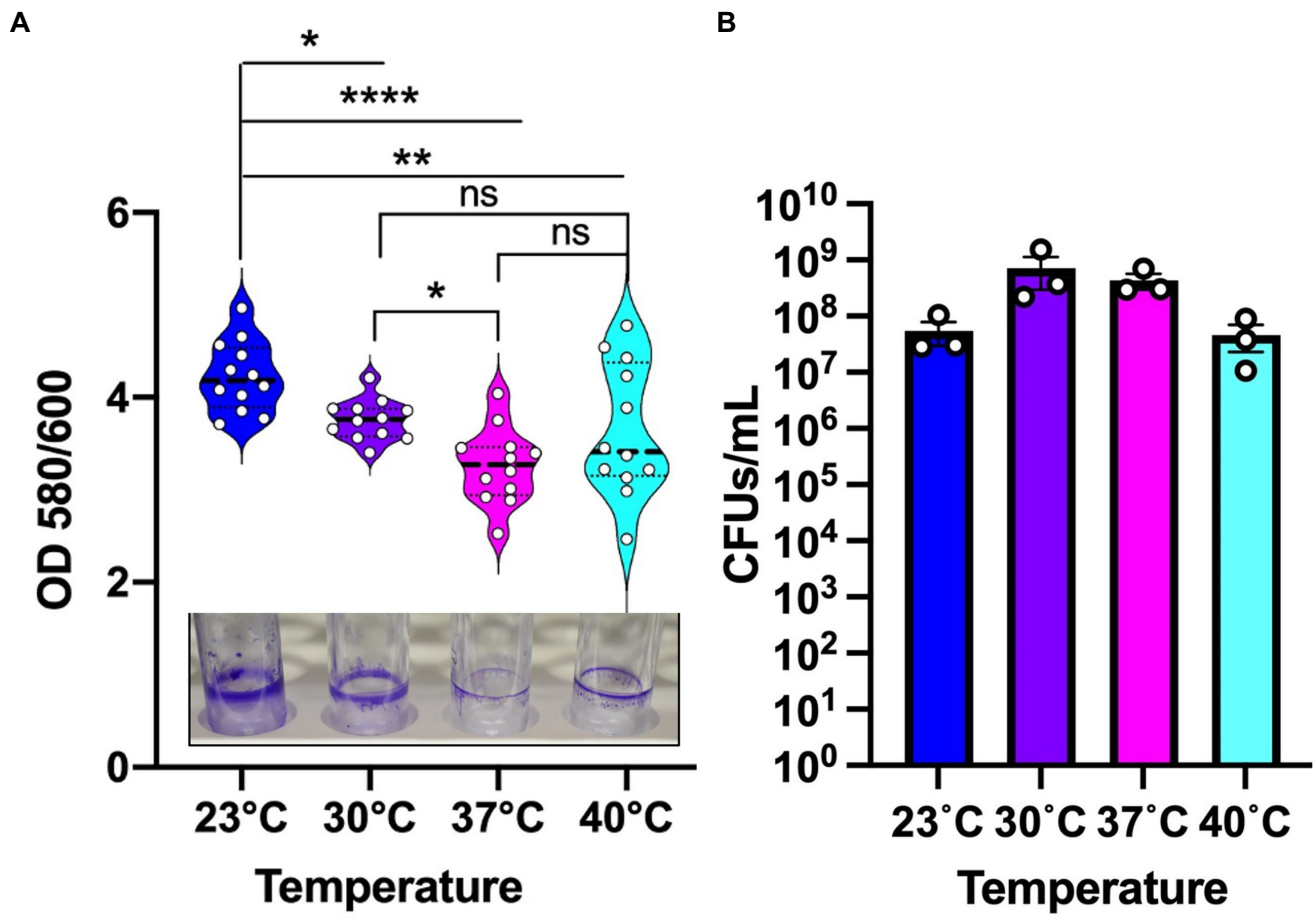
Environmental temperature biofilm is associated with higher biomass on polystyrene surfaces. **(A)** The biomass of the biofilm formed at the four temperatures. Violin plots represent means of three experiments with four replicates per sample. Crystal violet staining revealed subtle temperature-dependent architectural differences at the four temperatures. The center black line represents the interquartile range. Unpaired t-test (two-tailed) was used to measure statistical significance. ns: not significant, *p ≤ 0.05, **p ≤ 0.01, and ****p ≤ 0.0001. **(B)** CFU count of PA14 WT planktonic cells at environment versus host temperature. After 48 h of growth, the cultures were serially diluted in sterile 1X PBS and plated on LA plates. Plates were grown overnight at 37°C for sufficient colony formation. Error bars represent the standard error of mean of three biological replicates. The mean of each biological replicate was based on eight technical replicates after outlier analysis.

*Pseudomonas aeruginosa* is known to form biofilm in the different niches and undergoes a temperature shift when transitioning from an outside environment into the human body environment. To investigate the role of temperature in biofilm formation, we used *P. aeruginosa* lab strain UCBPP-PA14 (PA14) to grow biofilms at the four temperatures in a static condition. We used an established crystal violet (CV assay) staining method for biofilm and biomass measurement (O’Toole, 2011) and found that the biomass was higher at 23°C compared to the other temperatures (Figure 1A). As the temperature rose, there was a trend toward decreasing biofilm biomass. Of note, at 40°C we observed more variability in biofilm formation, with some replications displaying unusually high biomass and others displaying unusually low biofilm-associated biomass. This variation was consistent in all the rounds that we performed using the CV assay. Our observation of a higher biomass at lower temperature is consistent with the findings of other research groups (Townsley and Yildiz, 2015; Çam and Brinkmeyer, 2020; Kim et al., 2020).

To assess whether the temperature-dependent trends in biofilm formation were due to overall differences in growth, we measured viable cell numbers at 48 h for each temperature (Figure 1B) in addition to performing a full growth curve over this period (Supplementary Figure S1). These analyzes revealed that PA14 grows to the highest density at 30°C with 37°C providing the second most robust growth of this strain. The 40°C temperature is suboptimal compared to 30°C with 37°C, but 23°C resulted in the lowest amount of overall growth. Therefore, we can confidently state that overall growth levels achieved at the different temperatures are not the driver of the biofilm formation trends since 23°C and 30°C displayed the most robust biofilms even though 23°C displayed the lowest overall growth while 30°C displayed the highest overall growth. Thus, we conclude that the differences in overall biofilm at the different temperatures represent a true physiological adaptation to temperature shifts.

### Temperature-driven biofilm adaptations occur independently of nutrient sources and promote differential adherence to surface types

**Figure 2:**
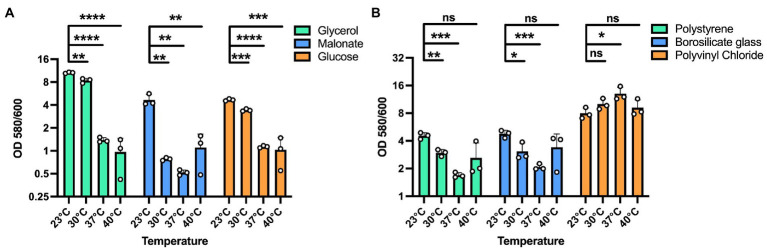
Effect of different substrates and materials on temperature-dependent biofilm formation. **(A)** The overall biofilm trend was same for all the carbon sources used. A 100 mM concentration of each carbon source in M9 media was used for biofilm growth at each temperature. In all cases, biofilm formation was most robust at 23°C and displayed decreasing levels with increasing temperatures. **(B)** The trend of higher biofilm formation at lower temperatures is observed on polystyrene and borosilicate glass. However, on polyvinyl chloride urinary catheters, biofilm formation was generally robust at all temperatures with a statistically significant advantage at 37°C. Bars represent the mean of three biological replicates performed on different days. The mean of each biological replicate was based on three technical replicates. Error bars represent the standard error of mean of the biological replicates. Unpaired t-test (two-tailed) was used to measure statistical significance. Y-axis is scaled by log2. ns: not significant, *p ≤ 0.05, **p ≤ 0.01, ***p ≤ 0.001, and ****p ≤ 0.0001.

We next sought to determine whether these temperature dependent biofilm growth trends were dependent on the nutrient composition of the media. Therefore, we tested temperature dependent biofilm formation in M9 minimal media with either glycerol, malonate, or glucose as the sole carbon source. All these growth substrates are known to play a role in influencing the metabolism of *P. aeruginosa* as reported in previous studies (Frimmersdorf et al., 2010; She et al., 2019; Elmassry et al., 2021). In all cases, the temperature-dependent biofilm trends were similar to those observed in LB (Figure 2A). Additionally, since *P. aeruginosa* can adhere to different surface materials, including both biotic and abiotic surfaces (Martin et al., 1993; Pericolini et al., 2018), we wanted to assess if differences in the surface material can have any impact on the biofilm formation at the four temperatures. For our experiments we used polystyrene and borosilicate glass material, the two most widely used materials for biofilm growth in lab environment as well as disposable urinary catheters made from polyvinyl chloride to represent a surface encountered in the clinic (Figure 2B). For both the polystyrene and borosilicate glass, we observed trends of more robust biofilm formation at environmental temperatures rather than host-associated temperatures. Interestingly, on the urinary catheters, overall biofilm biomass was greatly increased in all conditions with a slight biofilm growth preference observed at human body temperature. These results indicate that temperature adaptation in biofilms can occur independently of nutrient source and that the adaptations can lead to differential adherence properties to different substrates. Since EPS matrix is a vital component of the biofilm for surface adherence, we next wanted to study the temperature-specific EPS adaptations in *P. aeruginosa* biofilms.

### Biofilm architecture and EPS composition is modulated by temperature

**Figure 3:**
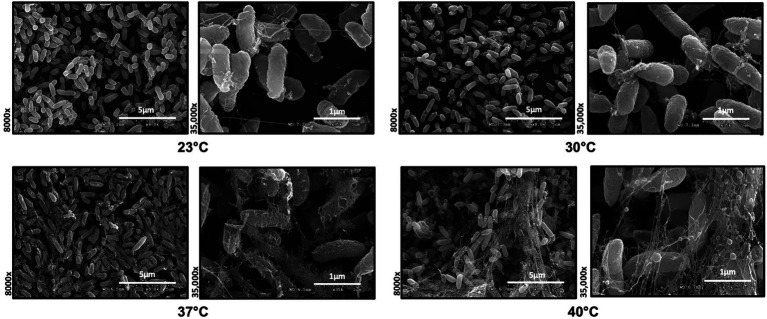
Microscopy reveals temperature-induced structural changes in *P. aeruginosa* biofilms. Comparison of the Scanning Electron Microscopy images of *P. aeruginosa* wild type biofilms grown for 48 h at 23°C, 30°C, 37°C and 40°C. Images show 8,000 Å ~ and 35,000 Å ~ magnification and are representative of three independent experiments.

To investigate the role of temperature on biofilm architecture, we first utilized microscopy tools. We grew *P. aeruginosa* strain PA14 biofilms at both environmental (23°C and 30°C) and host temperatures (37°C and 40°C) and visualized the biofilm using both scanning electron microscopy (SEM) and confocal laser scanning microscopy (CLSM). The SEM micrographs revealed a striking difference in EM-visible matrix production at environmental versus host temperatures. At 23°C and 30°C, the matrix appeared to be more scattered while at 37°C and 40°C, it was denser with cells entangled within the matrix (Figure 3). We also observed a few circular vesicle-like structures only in the 40°C biofilm (Supplementary Figure S2). Several studies have reported that high-temperature stress can change the composition of the outer membrane resulting in the formation of membrane vesicles (Cooke et al., 2019, 2020; Mozaheb and Mingeot-Leclercq, 2020). Also, a recent paper reported that explosive cell lysis in a subpopulation of cells under stress conditions could lead to the formation of membrane vesicles and biofilms (Turnbull et al., 2016). We believe these vesicle-like structures represent a similar phenomenon for our 40°C-grown biofilm.

**Figure 4:**
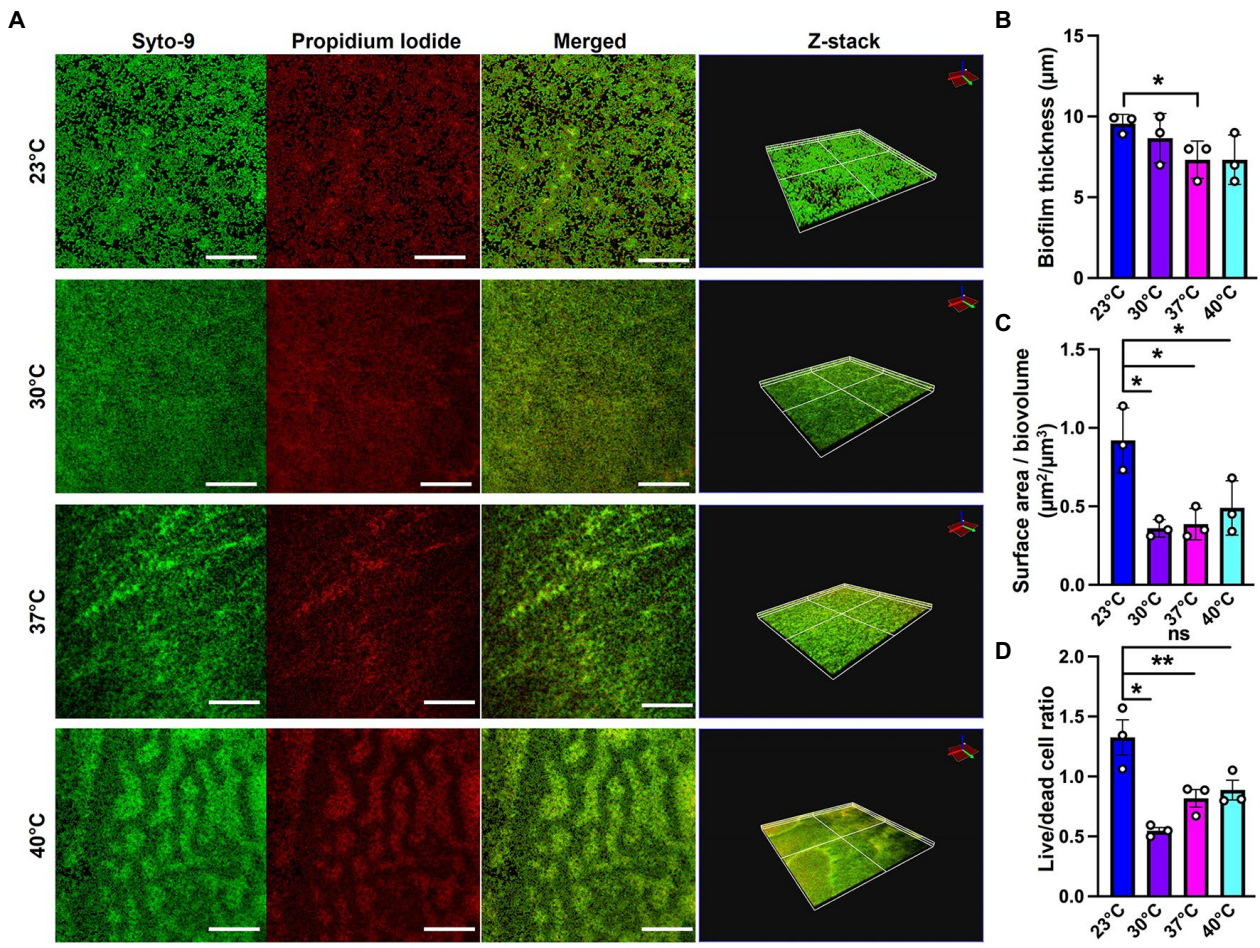
Microscopy using EPS-specific stains reveals temperature-induced structural changes in *P. aeruginosa* biofilms. **(A)** The live/dead staining results of *P. aeruginosa* PA14 biofilm after 48 h of growth at 23°C, 30°C, 37°C and 40°C. Biofilm was grown for 48 h on a microscope glass slide at 23°C, 30°C, 37°C and 40°C and stained with FilmTracer LIVE/DEAD Biofilm Viability kit; SYTO 9 shows live cells in green and propidium iodide shows dead cells in red. Scale bar: 50 μm. Z-stacks were collected for biofilms at the four temperatures using CLSM and analyzed by COMSTAT2 to determine. **(B)** The biofilm thickness and **(C)** the surface area to biovolume. **(D)** Live/dead cell ratio was analyzed using ImageJ. Images are representative of three independent experiments. Unpaired t-test (two-tailed) was used to measure statistical significance. ns: not significant, *p ≤ 0.05, and **p ≤ 0.01.

Since SEM data revealed higher levels of EM-visible EPS in glass-associated biofilms at higher temperatures even though we observed lower overall biofilm, we theorized that there are other components contributing to the robust biofilms forming at 23 and 30°C that might not be visible in our SEM micrographs, perhaps due to being washed away during sample processing steps. We therefore switched to CLSM to study the architecture of biofilms grown at different temperatures. To accomplish this, we used the Live/Dead Biofilm viability kit to observe the live versus dead cells as well as the overall biofilm architecture of *P. aeruginosa* at all the four temperatures (Deng et al., 2020). Also, since the propidium iodide (PI) which usually stains the dead cells, is known to stain the eDNA component of the matrix, we were able to observe some of the features of the matrix as well. Qualitatively, the overall architecture of the biofilm at 23°C and 30°C appeared slightly smoother with fewer textured features than biofilms grown at 37°C or 40°C (Figure 4A). On further quantifying certain parameters of the biofilm using COMSTAT2 and ImageJ, we observed the same trend of inverse correlation between biofilm thickness and temperature (Figure 4B). The surface area to biovolume ratio was also significantly higher in 23°C biofilms (Figure 4C). These trends did not appear to correlate with higher numbers of dead/lysed bacterial cells as the 23°C biofilms displayed the highest live to dead ratio of all the other conditions (Figure 4D). Therefore, it is likely not eDNA released from lysed cells that is contributing to the robust biofilms at 23°C.

**Figure 5:**
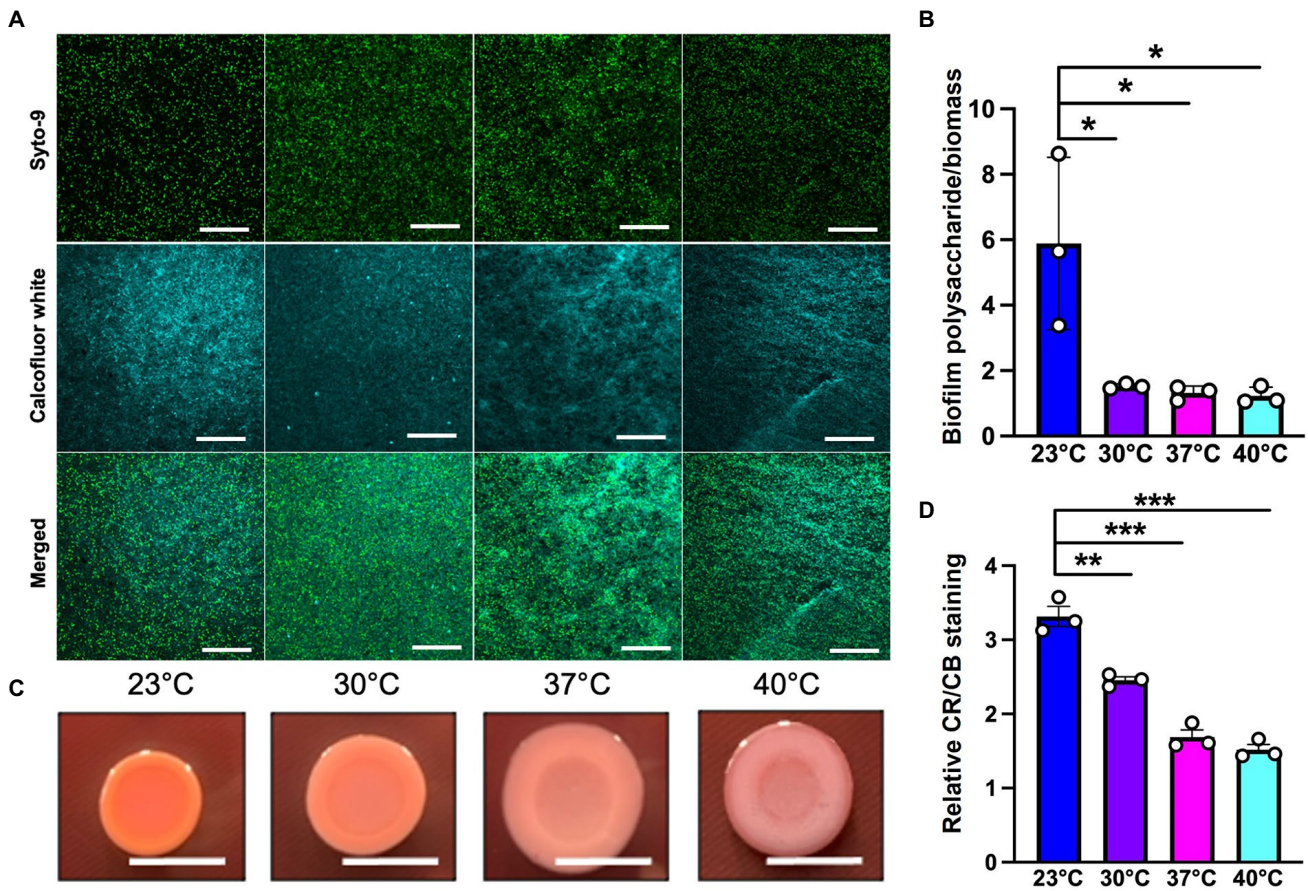
Microscopy using EPS-specific stains reveals temperature-induced increase in EPS carbohydrates in *P. aeruginosa* biofilms grown at 23°C. **(A)** The biofilm staining results of *P. aeruginosa* PA14 biofilm after 48 h of growth at 23°C, 30°C, 37°C and 40°C. Biofilm was grown for 48 h on a microscope glass slide at 23°C, 30°C, 37°C and 40°C and stained with SYTO 9 (live cells in green) and Calcofluor white (stains the polysaccharide component of the matrix cyan). Scale bar: 50 μm. **(B)** Quantification of biofilm polysaccharide per biomass in *P. aeruginosa* PA14 biofilm after 48 h of growth at 23°C, 30°C, 37°C and 40°C using ImageJ. **(C)** Congo red binding assay reveals temperature specific difference in colony morphology. Extracellular matrix production by the wildtype was evaluated on tryptone agar plates containing Congo Red and Coomassie brilliant blue G after incubation at the four temperatures for 72 h. Representative images of the colony morphologies of PA14 are shown. Scale bar: 1 cm. **(D)** Quantification of CR and CB stain was performed using ImageJ. CR = Congo Red and CB = Coomassie Blue. Unpaired t-test (two-tailed) was used to measure statistical significance. *P≤0.05, **P≤0.01, and ***P≤0.001.

Since polysaccharide is one of the important components of the extracellular matrix, we next wanted to investigate its presence at different temperatures. For this purpose, we used calcofluor white, a polysaccharide-binding dye, which has been previously used to visualize the extracellular matrix (Neut et al., 2005; Wakeman et al., 2016; Figure 5A). This dye can bind to a variety of polysaccharides, with a strong interaction with glycosidic bonds, mainly β1-3 and β1-4 polysaccharide (Alemayehu et al., 2012; Soler-Arango et al., 2019). On quantifying the polysaccharide content relative to overall biofilm cell density, we observed that the room temperature biofilm had a slightly higher polysaccharide content per cell compared to the rest of the temperatures (Figure 5B). Further support of the temperature-associated changes in polysaccharide content in the EPS was provided by growth on Congo Red plates (Figure 5C; Madsen et al., 2015). This assay utilizes a Congo Red dye that binds to the polysaccharide component of the matrix and a Coomassie Blue dye that binds to the protein component of the matrix. The PA14 colonies looked morphologically different at all the four temperatures, with greater apparent Congo Red dye absorption and less apparent Coomassie Blue dye absorption at 23°C compared to the other three temperatures (Figures 5C,D). Previous work by our group has shown that there is a global proteomic difference in biofilms associated with 23°C and 37°C temperatures (Bisht et al., 2021), which further bolsters our present finding where we observe a different level of Coomassie dye absorption by the matrix at all four temperatures. Finally, the results in this study are consistent with previous findings stating the relation between temperature and polysaccharide production (Frølund et al., 1996; Sakuragi and Kolter, 2007). In total, these findings suggest that the polysaccharide component of the matrix could be contributing to biofilm formation at room temperature.

Previous studies have observed that pel and alginate carbohydrates genes increase at 20–23°C relative to 37°C in *P. aeruginosa* (Sakuragi and Kolter, 2007; Kim et al., 2020). Therefore, it is possible that this phenomenon is occurring in our biofilms. However, we did not observe any striking differences in gene expression for pel or alginate biosynthetic genes (Supplementary Table S1) when we analyzed our previously published RNA sequencing data set (Bisht et al., 2021). However, these data do not exclude the possibility that temperature changes are influencing protein and/or activity levels of these polysaccharides. Therefore, we directly assessed the biofilm forming capacity of mutants lacking *pelB* and *algD* to demonstrate that neither pel nor alginate serve to specifically bolster biofilms grown at lower temperatures. The *pelB* mutant was found to be generally defective at all temperatures, while the *algD* mutant was generally expendable at all temperatures (Supplementary Figure S3). This finding highlights the general rather than temperature-specific importance of pel as an essential component of the matrix in the PA14 *P. aeruginosa* strain. Overall, our results are indicative of the presence of more EM-visible matrix and greater biofilm texture at higher temperatures and a flatter but generally thicker biofilm architecture at lower temperatures. We also see a slightly higher polysaccharide content at lower temperature, consistent with the findings of some other research groups, which could be contributing toward the higher biofilm biomass observed at this temperature. However, future work focusing on the characterization of polysaccharide components at low temperature biofilm is required, especially since no temperature-specific polysaccharide requirements were identified for PA14.

### Genetic requirements to establish a robust biofilm at environmentally relevant temperatures

Because specific components contributing to the robust biofilm formation at room temperature remained elusive, a preliminary screening was performed on a commercially available transposon mutant library of *P. aeruginosa* comprising of over 5,500 mutant genes (Liberati et al., 2006) to identify temperature specific genetic requirements. We performed the established microtiter dish biofilm formation (crystal violet) assay to assess biofilm biomass of wild type and mutants at all the four temperatures (O’Toole, 2011). While most of the potential hits in this screen remain to be validated and thus are not included in the present manuscript, we turned our focus to hypothetical/uncharacterized proteins that might be specifically contributing to biofilm growth and adaptation at 23°C to better understand the odd phenomenon of temperature specific biofilm increases with no currently characterized genetic contributors to this structure.

**Figure 6:**
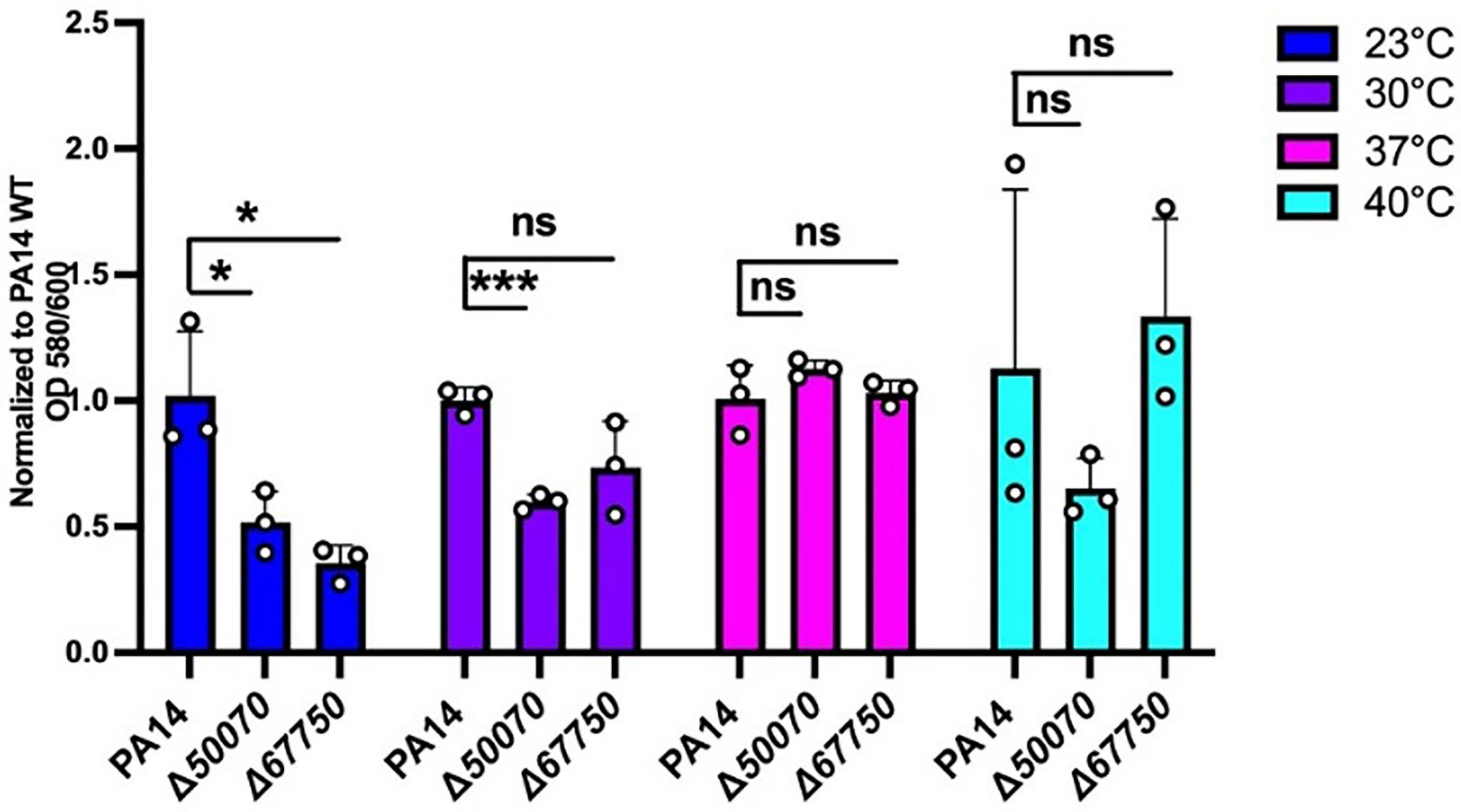
Growth requirements for biofilm formation at environmental versus host temperatures. The biofilm biomass (ratio of OD580/OD600), at each temperature for the two mutants was normalized relative to WT PA14 at the different temperatures. The biomass of the biofilm was defective at 23°C for both mutants and at 30°C for PA14_50070 when compared to PA14. Bars represent the mean of three biological replicates performed on different days. The mean of each biological replicate was based on three technical replicates. Error bars represent the standard error of mean of the biological replicates. Unpaired t-test (two-tailed) was used to measure statistical significance between the wildtype and each mutant. ns: not significant, and *p ≤0.05, and ***p ≤0.001.

The mutants in genes *PA14_50070* and *PA14_67750* were among the hits showing a significant reduction in biofilm formation at 23°C when compared to wild-type (Figure 6; Supplementary Figure S3). *PA14_50070* which encodes for a hypothetical protein showed a defect in biofilm formation at 23°C and 30°C. Further characterization of this protein in the future can help us gain some more information about its role in low-temperature biofilm formation and adaptation. Another mutant of gene *PA14_67750*, encoding for a rhodanese-like domain-containing protein, also showed a defective biofilm: biomass ratio at 23°C and a trend toward a defect at 30°C (Figure 6; Supplementary Figure S3). Because this gene is found in an apparent operon, we also performed the *PA14_67750* complementation to confirm if the defect was due to the *PA14_67750* gene and not the *PA14_67740* (*grx*) gene that lies next to it. Our complementation experiment was able to recapitulate our findings, thus strengthening our observation that *PA14_67750* indeed plays a key role in biofilm formation specifically at 23°C (Supplementary Figure S4). Additionally, the Congo red binding assay showed subtle changes in the colony morphology between the WT and the *PA14_50070* and *PA14_*67750 deletion mutants (Supplementary Figure S5). For example, there is a trend toward a slight decrease in the level of Congo Red staining in these strains at 23°C and 30°C. We therefore believe that these hypothetical proteins represent previously uncharacterized temperature-specific contributors to *P. aeruginosa* biofilm formation and could be potential gene targets to eradicate biofilms growing at environment-associated temperature.

Since *P. aeruginosa* PA14 uses different strategies to form biofilms than other strains of *P. aeruginosa*, such as not having the psl operon, PA14 is considered somewhat of an outlier in species group. For this reason, we wanted to explore the relative conservation of our genes of interest. We downloaded all of the completed *P. aeruginosa* strains from NCBI database (accessed October 2022), 494 in total and ran them through Roary (Page et al., 2015) to assign the genes to ortholog groups. We found that *algD* and *pelB* were found in 98.78 and 36.03% of the strains analyzed, respectively. Conversely, all strains contained a gene which was assigned to the ortholog groups containing either *PA14_50070* and *PA14_67750*, (Supplementary Table S2). Additionally, we observed potential synteny or conservation of the sequence of genes in the region surrounding both genes. (Supplementary Figure S6) As a result of this data, we believe that our observations made regarding both hypothetical proteins to be relevant to the species group as a whole. However, future studies will be required to further verify this contention.

## Conclusion

Temperature is an important external stimulus that can tremendously impact the biofilm formation changing both gene and protein expression, as well as the metabolite levels (Bisht et al., 2021; Li et al., 2022). In this study we wanted to observe the effect of temperature on biofilm architecture at both environment and human host associated temperatures. We also sought to discover genes contributing to novel *P. aeruginosa* biofilm adaptations at room temperature rather than human body temperature. Overall, the data presented herein indicate that temperature can induce EPS-specific adaptations in *P. aeruginosa* by affecting the matrix composition and promoting differential surface association. We also report that despite an apparent increase in biofilm carbohydrates at 23°C, no known carbohydrates of strain PA14 were specifically required for biofilm formation at this temperature, with pel being generally required and alginate being generally expendable. Instead, previously uncharacterized hypothetical proteins appear to be playing a role in the temperature-specific biofilm adaptation. An in-depth understanding of the biofilm matrix of *P. aeruginosa* in its varied habitats is thus essential. By finding drug targets specific to particular niches (environmental versus host), we may be able to limit the spread of antibiotic resistance *via* environmentalreservoirs.

## Materials and methods

### Bacterial strains, media, and growth conditions

*P. aeruginosa* strain UCBPP-PA14, a highly virulent strain of *P. aeruginosa* originally isolated from a wound infection, was used in all experiments unless otherwise stated (He et al., 2004). Mutants used for the screening are mentioned in Table 1 (Liberati et al., 2006). Strains were routinely grown overnight and maintained at 37°C in Luria-Bertani (LB) broth. Gentamicin was added at 15 ug/ml to maintain the transposon in the mutants. Also, 100 mM of glucose, malonate, and glycerol in M9 media were used to assess biofilm biomass.

**Table 1:** Mutants selected to assess their role in biofilm formation at the four temperatures.

PA14 with *MAR2xT7 mariner* transposon insertion within the specific gene; Gm^r^ (Liberati et al., 2006)			
Gene locus	Gene name	Product description	Functional category
PA14_18580	*algD*	GDP-mannose 6-dehydrogenase AlgD	Secreted Factors (toxins, enzymes, alginate)
PA14_24490	*pelB*	Hypothetical protein	Hypothetical, unclassified, unknown
PA14_50070	Hypothetical protein	Hypothetical protein	Hypothetical, unclassified, unknown
PA14_67750		Rhodanese-like domain-containing protein	Transport of small molecules

### Crystal violet assay

To quantify and study biofilm formation at each temperature we used the previously established microtiter biofilm formation assay (O’Toole, 2011). Wild-type *Pseudomonas aeruginosa* PA14 and the mutant strain were grown overnight in a 96 well round bottom plate in 150 microliters of LB broth with shaking at 220 rpm and 37°C overnight incubation. Next day, five microliters of the overnight culture were transferred to a fresh 96 well plate with 145 μl of LB media. This was done for 3 replicates at 3 different days. The plates were incubated for 48 h at 23°C, 30°C, 37°C and 40°C. An absorbance reading at 600-nm wavelength was taken after 48 h using a Synergy Hi5 Microplate Reader, Biotek. Planktonic cells were then aspirated out and the remaining biofilm was washed three times with 300 microliters of PBS. This step helps remove unattached cells and media components that can be stained in the next step and significantly lowers background staining. Next, 200 microliters of 100% ethanol were added to the wells and incubated for 15 min. The ethanol was then aspirated out completely and the plates are flipped upside down and left for drying. After the ethanol dried, 200 μl of a 1% solution of crystal violet (CV) was added to each well of the microtiter plate. The microtiter plate was then incubated for 15 min at room temperature followed by rinsing it 3–4 times with water by submerging the plate in a tub of water and blotting vigorously on a stack of paper towels to get rid of the excess water. The microtiter plate was then left to dry for 1–2 h. Finally, 150 microliters of 30% acetic acid solution were added to each well of the microtiter plate to solubilize the crystal violet. After an hour incubation at room temperature, an absorbance reading was taken at 580 nm. Using the biomass baseline, this reading was quantified and analyzed to produce readable data. For data comparing biofilm formation on polystyrene versus borosilicate glass, cells were grown in 5 ml culture tubes composed of these two substrates each containing 0.5 ml of culture. Sample processing mimicked that of the microtiter plates. Prior to analysis, outliers were removed by determining the Q1 and Q3 values of each test group and then values outside of 1.5 times the range of Q1 and Q3 were removed.

### Biofilm formation on urinary catheters

Four overnight PA14 wild type (WT) isolates on LA plates were inoculated into 200 μl of LB media in a 96-well round bottom plate and grown overnight at 37°C. 145 μl of LB media was added into eight wells in a 96-well plate and 5 μl of each overnight culture was added into 2 of those wells. One of the duplicates acted as a control (no catheter) and to another, a 1 cm long piece of sterile urinary catheter (Cure Medical) was added. This inoculation was repeated three more times, so that there would be four plate copies: one for each temperature (23°, 30°, 37° and 40°C). Plates were placed in their respective incubators for 48 h. The fixing, staining and OD readings mentioned in “Crystal Violet Assay” method was followed with minor changes. After fixing and staining, the catheters were moved to clean wells in between each DI wash and the solubilized CV in acetic acid from the catheter wells had to be diluted to 1:10 ratio prior to taking the OD580 reading.

### Bacterial growth curve

Frozen cultures of mutants and PA14 WT were streaked out on LA plates and incubated overnight at 37°C. Isolated colonies were selected from both the WT and mutants which were then inoculated in 200 μl of LB within a 96-well round bottom plate. The plate was incubated for 18 h at 37°C in shaking conditions. In a 96-well flat bottom plate, 145 μl of LB and 5 μl of overnight grown cultures were added into each well. This step was repeated 3 more times to have a plate for each temperature (23°, 30°, 37° and 40°C). The 0-h OD 600 reading was taken, and plates were placed into their respective static incubators. Additional OD 600 readings were taken at 2, 6, 12, 24, 30, 36, and 48 h. Blank LB was used for background subtraction. The protocol was done on two independent days. The OD readings for all the time points were plotted using GraphPad Prism 8.

### Cell viability assay

A frozen culture of PA14 wile type (WT) was streaked on an LA plate and grown at 37°C overnight. Eight isolated colonies were picked and inoculated into 200 μl of LB within a 96-well round bottom plate. The culture plate was incubated for 18 h at 37°C in shaking conditions. 5 μl of overnight culture was inoculated into 145 μl of fresh LB within a 96-well flatbottom plate. This step was done a total of 4 times, one plate for each temperature (23°, 30°, 37° and 40°C). The plates were placed in their respective incubators in static conditions for 48 h. Afterwards, 1:10 serial dilutions of the cultures were done in filtered sterilized 1X PBS (a total of eight dilutions steps were needed). 5 μl of each dilution was spot plated onto LA plates and incubated overnight at 37°C. This was repeated on three independent replicates. Once all of the CFUs were counted, replicates were subjected to outlier analysis as mentioned previously and the averages of each independent replicate was calculated, which were plotted using GraphPad Prism 8.

### Congo red binding assay and quantification

To phenotypically assess the different components of the EPS matrix, a colony-morphology assay was performed as described previously (Madsen et al., 2015). Extracellular matrix production by *P. aeruginosa* PA14 and the mutants was evaluated on tryptone agar plates containing Congo Red and Coomassie brilliant blue G after incubation at 23°C, 30°C, 37°C and 40°C for 24, 48 and 72 h. Five microliters of overnight precultures were spotted on Petri plates containing 20 ml of the assay medium (1% tryptone, Congo red dye (40 μg/ml), Coomassie brilliant blue G dye (20 μg/ml) and 1% agar). Colonies were grown at 23°C, 30°C, 37°C and 40°C for 24, 48 and 72 h. Images of the colonies were taken daily using a Nikon camera. For quantification purpose, the images were first imported into ImageJ, after that the red (Congo red) and blue (Coomassie blue) channels were used to calculate the area and mean intensity. Normalization of all the mean integrated density values were done with respect to the area and the background. Apparent Congo red to Coomassie blue ratio was then calculated and plotted using GraphPad Prism.

### Electron microscopy

For scanning electron microscopy (SEM), static cultures were grown in 50-ml conical tubes with circular glass coverslips semi-submerged in 2 ml of LB broth. Coverslip samples were handled and processed as described previously (Gaddy et al., 2009). Upon removal of the culture medium, the coverslips were immediately flooded with 2.5% glutaraldehyde in 0.05 M sodium cacodylate and incubated at room temperature for 1 h. The fixative was then removed and replaced immediately with 0.05 M sodium cacodylate to prevent sample dehydration. Next, the coverslips were incubated in osmium tetroxide for 15 min followed by dehydration with increasing concentrations of ethanol, ranging from 25 to 100%, and CO_2_ critical point drying. Samples were carbon-coated and visualized with a Hitachi S-4300 scanning electron microscope.

### Biofilm imaging and quantification

Biofilms grown on glass slides for 48 h were imaged by confocal laser scanning microscopy (CLSM) (Olympus; FV3000). 20 μl of the overnight grown culture of PA14 were added into a fresh 20 ml of LB broth, in a 50-mL Falcon tube along with a microscope glass slide, and grown for 48 h. The biofilm was then washed with PBS followed by staining with FilmTracer LIVE/DEAD Biofilm Viability kit (ThermoFisher). Next, biofilm was incubated with SYTO™ 9 and propidium iodide stains for 20 min in the dark at room temperature as per the manufacturer’s protocol. After that, the biofilm was washed with PBS and the slides were imaged using a OlympusFV3000 microscope with an objective lens of ×60 (oil). For another experiment, to visualize bacterial cells we used SYTO™ 9 and to visualize polysaccharide component of the matrix, calcofluor white (blue; Sigma-Aldrich) was used. For each glass slide, five image stacks were taken with a z-step size of 0.7 μm. Biofilm quantification at all four temperatures were done using COMSTAT2 plugin in ImageJ (Heydorn et al., 2000). ImageJ was used to quantify the live/dead cell ratio. Images were analyzed by first splitting each image into RGB channels and then collecting the fluorescence integrated density values for SYTO™ 9 stain from the green channel and propidium iodide from the red channel. Normalization of all the fluorescence integrated density values were done with respect to the area and the background. Finally, a ratio of SYTO™ 9 relative to propidium iodide signal was calculated using the average density values of each image and plotted. Similarly, ImageJ was also used to quantify polysaccharide/biofilm biomass content for which images were analyzed by first splitting each image into RGB channels and then collecting the fluorescence integrated density values for calcofluor white stain from the blue channel and for SYTO™ 9 stain from the green channel. Normalization of all the fluorescence integrated density values were done with respect to the area and the background. Finally, a ratio of calcofluor white signal relative to SYTO™ 9 signal was calculated using the average density values of each image and plotted using GraphPad Prism. Each experiment included three independent biological replicates, and at least three images were taken for each replicate.

### Plasmid construction

To design constructs for the complementation assay, we used the previously established method with slight modifications (Hmelo et al., 2015). First, DNA was extracted from PA14 WT overnight grown culture, followed by amplification of the gene loci *PA14_67750* using forward and reverse primers. The primers were designed for *PA14_67750* with minor modifications upstream of the gene to add HindIII and EcoRI restriction sites. The primers *PA14_67750*-F (5′-CCGGCAGAATTCCGCCCTGGATATCCGTCACC-3′) and *PA14_67750*-R (5′-GATCACAAGCTTCGGCATGTCACTTCACCAGC-3′) targeting *PA14_67750* were designed for our experimental use. PCR was conducted using the Q5 High-Fidelity Master Mix (New England BioLabs) followed by PCR purification (QIAGEN PCR purification kit). pHERD20T-specific primer sequences were obtained from a previously published study (Qiu et al., 2008). Isolated pHERD20T plasmid and the purified PCR products were digested by HINDIII and EcoRI, followed by ligation of the digested products using a quick ligation mix (New England BioLabs). The ligated products were then chemically transformed into *Escherichia coli* S17 and incubated on Luria Agar (LA) [with carbenicillin (50 μg/ml)] plates. Isolated colonies were selected, and plasmid was confirmed by colony PCR and gel electrophoresis. Confirmed colonies were grown in 5 ml of LB at 37°C overnight and stored. Transposon mutants ∆*PA14_67750* was streaked on LA plate and the *E. coli* S17 mutant was grown on LA [with carbenicillin (50 μg/ml)] at 37°C overnight. Isolated colonies were grown in 5 ml of LB at 37°C (carbenicillin was added to *E. coli* S17 mutants to have a final concentration of 50 μg/ml). 1 ml of the overnight cultures of *E. coli* S17 mutant (donor) was pelleted, and supernatant was discarded. Simultaneously, 1 ml of ∆*PA14_67750* (recipient) was added to the donor pellet and resuspended. Next, 400 μl of the donor/recipient mixture and donor only mixture was spotted on LA plates and grown for 5 h at 37°C. The spotted cultures were collected and suspended in 1 ml of sterile 1X PBS, pelleted and washed with 1 ml of sterile 1X PBS. 1:10 dilutions of conjugates were plated on Vogel-Bonner minimal medium (VBMM) salt plates [with carbenicillin (300 μg/ml)] and grown at 37°C overnight. Overnight cultures were then streaked on LA [with carbenicillin (300 μg/ml)] plates to get isolated colonies and grown at 37°C. Colonies were selected and constructs were confirmed by colony PCR and gel electrophoresis. Confirmed colonies were grown in 5 ml of LB [with carbenicillin (300 μg/ml)] overnight at 37°C and stored.

### Biofilm assay using complementation construct

Tn-*PA14_67750::67750*, Tn-*PA14_67750*:pHERD, and PA14 WT:pHERD was streaked on LA [with carbenicillin (200 μg/ml)] plates and grown overnight at 37°C. Isolated colonies from each plate were selected and inoculated in a 96-well plate with 200 μl of LB [with carbenicillin (200 μg/ml)]. The plate was grown at 37°C overnight in shaking conditions. Next day, 145 μl LB [with carbenicillin (200 μg/ml) and 1.5% arabinose] was added into wells of four sterile 96-well plates and then inoculated with 5 μl of overnight culture. Each plate was wrapped in plastic wrap and placed at 23°C in dark and grown for 48 h. Previously established microtiter biofilm formation assay, as discussed earlier, was then performed. Each experiment included four independent biological replicates and was performed on three different days.

### Orthologous analysis of *Pseudomonas aeruginosa* strains

The FASTA files of the complete genomes for *P. aeruginosa* were downloaded from NCBI. The genomes were ran through Prokka software to annotate (Seemann, 2014). The annotated GTF files were then compared for orthologs using the default settings of Roary (Page et al., 2015). The output file “gene_presence_absence.csv” was used to determine the number of genes by strain to each ortholog group.

### Statistical analysis

Statistical analyses were performed using GraphPad Prism 8.0 (GraphPad Software, Inc., San Diego, CA). Unpaired t-test (two tailed) was used to calculate the statistical significance.

## Data availability statement

The original contributions presented in the study are included in the article/Supplementary material, further inquiries can be directed to the corresponding author.

## Author contributions

KB contributed to the project design, data acquisition, interpretation of data, and manuscript writing. AL contributed to the complementation assay, data acquisition and interpretation of the biofilm assay as well as manuscript editing. CW contributed to project design, data acquisition, interpretation of data, and editing of the paper. All authors contributed to the article and approved the submitted version.

## Funding

This research work in the Wakeman lab was supported by NIH/NIGMS (R15GM128072). KB was supported by the Doctoral Dissertation Completion Fellowship granted from Texas Tech University Graduate School. CAW received publication funding through TTU Open Access Fund. ARL received publication funding through TechASM.

## Conflict of interest

The authors declare that the research was conducted in the absence of any commercial or financial relationships that could be construed as a potential conflict of interest.

## Publisher’s note

All claims expressed in this article are solely those of the authors and do not necessarily represent those of their affiliated organizations, or those of the publisher, the editors and the reviewers. Any product that may be evaluated in this article, or claim that may be made by its manufacturer, is not guaranteed or endorsed by the publisher.
