# Supplementary material for: Temperature-specific adaptations and genetic requirements in a biofilm formed by Pseudomonas aeruginosa
Source: Front Microbiol. 2023 Jan 6;13:1032520. doi: 10.3389/fmicb.2022.1032520 (PMC9853522; doi:10.3389/fmicb.2022.1032520)
Supplement: Supplementary file 1 [file Data_Sheet_1.pdf]

Supplementary information

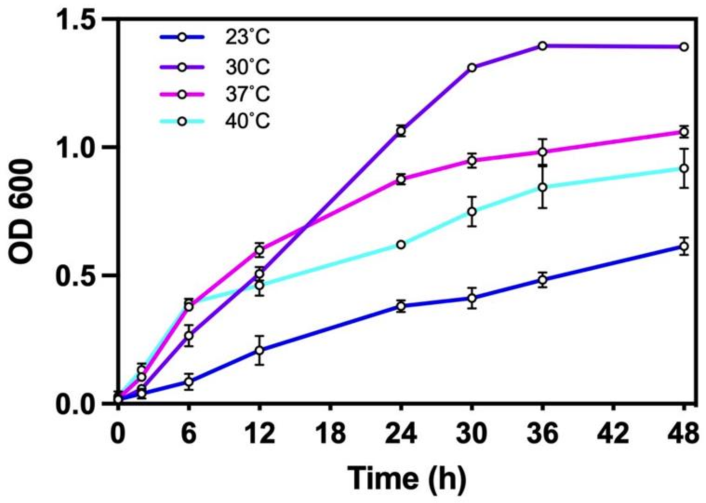

Supplementary Figure 1. Growth curve of PA14 WT planktonic cells at environment versus host temperature. OD600 measurements were taken at different time points until 48 hours of growth period for planktonic cells grown at the four temperatures. Error bars represent the standard error of mean of two biological replicates. The mean of each biological replicate was based on eight technical replicates after outlier analysis.

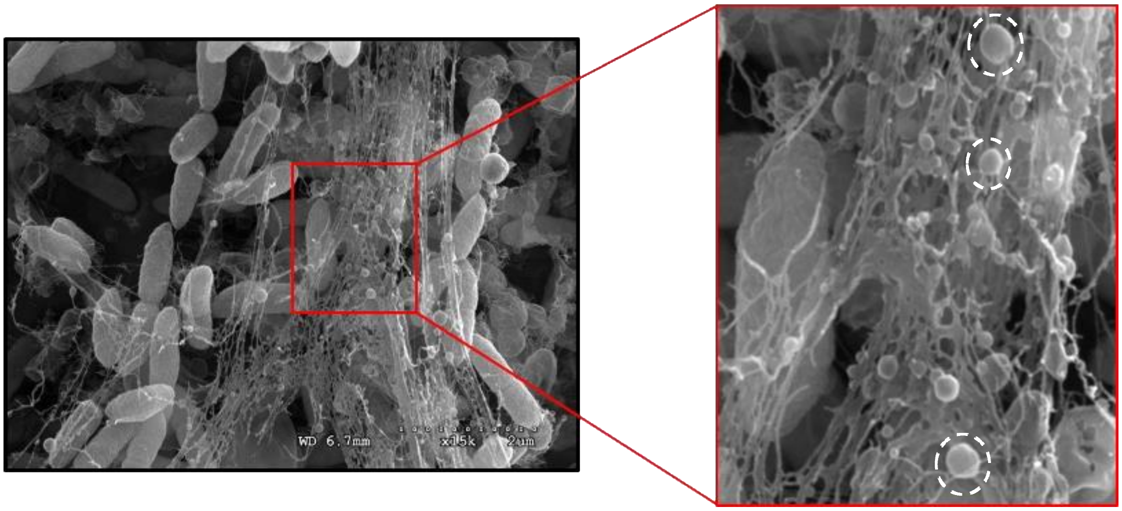

Supplementary Figure 2. Zoomed in image of potential OMVs which are observed in 40°C grown biofilms. The dashed white circles highlight some of the OMVs.

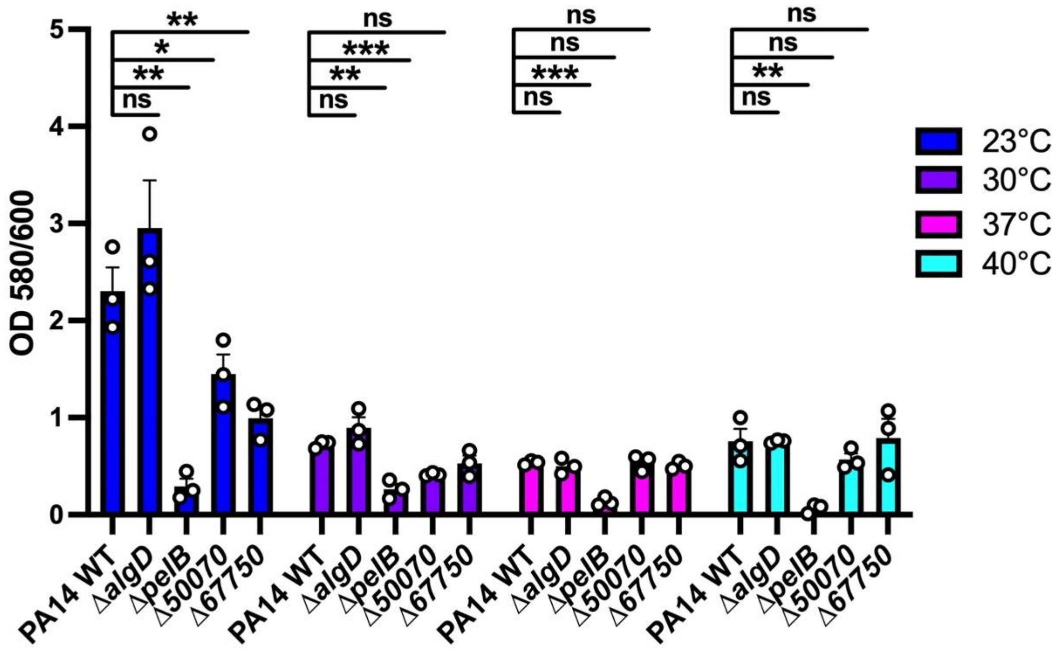

Supplementary Figure 3. Biofilm biomass measurement at 48 hours of growth period for PA14, Δ*algD*, Δ*pelB*, Δ*PA14_50070*, and Δ*PA14_67750*. Error bars represent the standard error of mean of three biological replicates performed on different days. The mean of each biological replicate was based on four technical replicates. Unpaired t-test (two-tailed) was used to measure statistical significance between the wildtype and each mutant. ns: not significant, *P≤0.05, **P ≤ 0.01., ***P ≤ 0.001

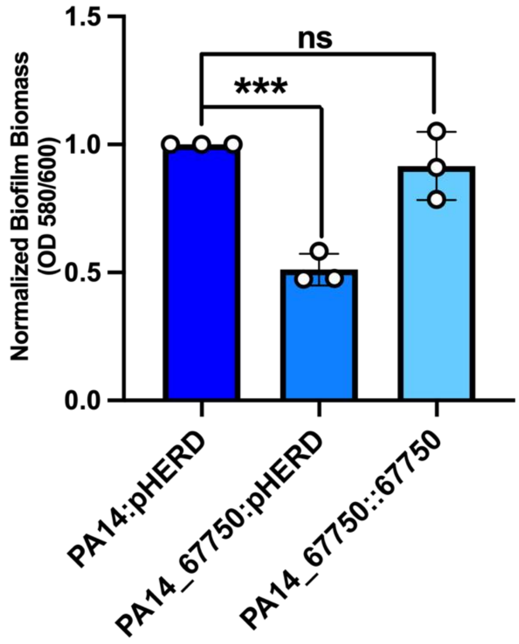

Supplementary Figure 4. Biofilm biomass measurement at 48 hours of growth period for PA14:pHERD, PA14_67750:pHERD and PA14_67750::67750 was performed at 23˚C. Biofilm biomass of the mutant was normalized to the PA14:pHERD readings. Error bars represent the standard error of mean of three biological replicates performed on different days. The mean of each biological replicate was based on four technical replicates. Unpaired t-test (two-tailed) was used to measure statistical significance between the wildtype and each mutant. ns: not significant, ***P ≤ 0.001.

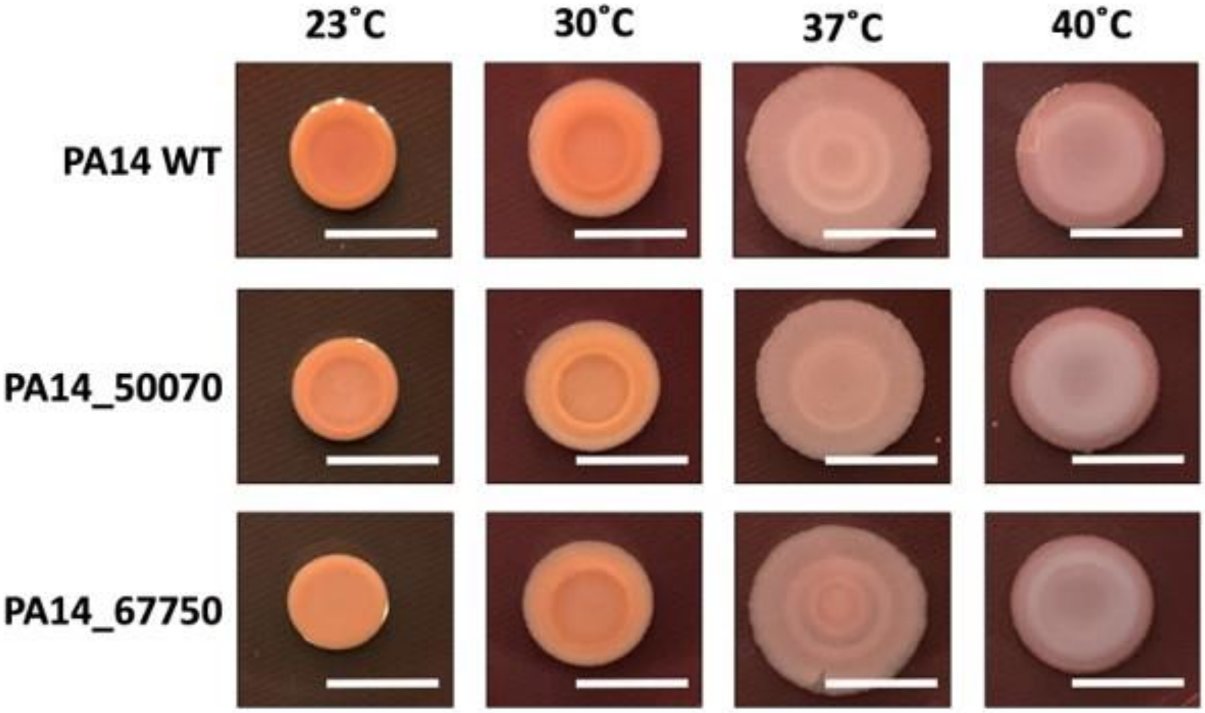

Supplementary Figure 5. Congo Red Assay of PA14 WT and selected mutants after 72 hours. Comparison of PA14 WT to transposon mutants on Tryptone Agar containing Congo Red and Coomassie Brilliant Blue G grown at four different temperatures. The spots are representative of three independent rounds Scale bar: 1 cm.

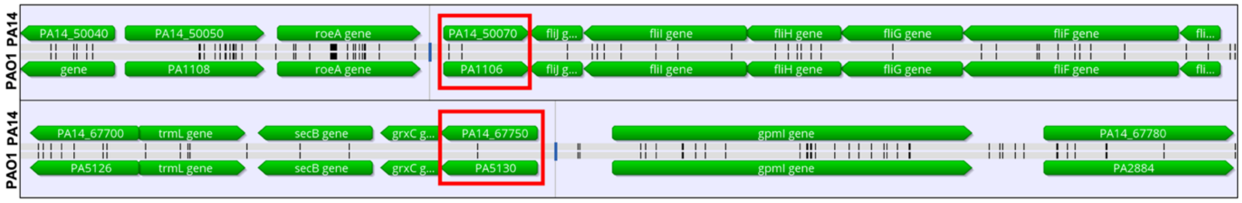

Supplementary Figure 6. Observed synteny for the region around PA14_50070 and PA14_67750. The genomes were downloaded from NCBI. Alignment was done using Geneious Prime. The alignment matches the order of orthologous groups in the Roary data for all strains we viewed. PA14 and PAO1 strains (top and bottom genomes respectively) were selected for the purposes of this illustration.

Supplementary Table 1: List of genes associated with pel and alginate polysaccharides regulated by temperature in biofilm state. A ratio of log2 fold change in the gene expression at 23˚C versus 37˚C in the biofilm growth state, along with the p-value and q-value has been shown for each gene.

Name	Locus	Fold-change Log2(B23/B37)	P-value	Q-value
pelA	PA14_24480	-0.61	7E-06	3E-05
pelB	PA14_24490	-0.60	2E-06	1E+00
pelC	PA14_24500	-0.77	2E-09	1E+00
pelD	PA14_24510	-0.33	5E-04	1E+00
pelE	PA14_24530	-0.67	1E-08	1E+00
pelF	PA14_24550	-0.45	1E-05	1E+00
pelG	PA14_24560	-1.26	1E-25	1E+00
algA	PA14_18380	-0.58	3E-04	1E+00
algF	PA14_18410	-1.00	4E-02	1E+00
algJ	PA14_18430	0.00	9E-02	1E+00
algI	PA14_18450	-0.42	2E-05	1E+00
algL	PA14_18470	-1.00	5E-06	1E+00
algX	PA14_18480	-0.42	2E-04	1E+00
algG	PA14_18500	-0.68	1E-08	1E+00
algE	PA14_18510	-2.00	2E-19	1E+00
algK	PA14_18520	-1.00	6E-06	1E+00
alg44	PA14_18550	-0.58	7E-04	1E+00
alg8	PA14_18565	-1.14	1E-16	1E+00
algD	PA14_18580	-1.42	1E-26	1E+00
algU	PA14_54430	-1.37	2E-09	8E-09
algW	PA14_57760	-0.43	6E-04	2E-03
algP	PA14_69370	0.35	5E-01	1E+00
algQ	PA14_69390	-0.06	3E-02	8E-02
algR	PA14_69470	0.43	5E-01	1E+00
algZ	PA14_69480	-0.56	2E-05	6E-05
algC	PA14_70270	0.29	7E-01	1E+00
algB	PA14_72380	-0.43	7E-05	1E+00

Supplementary Table 2: List of genes pulled from Roary ortholog data.

Gene Name	Gene Locus in PA14	Annotation	Ortholog Conservation (%)
	PA14_50070	Hypothetical protein	494/494 (100%)
	PA14_67750	Hypothetical protein	494/494 (100%)
algD	PA14_18580	GDP-mannose 6-dehydrogenase	488/494 (98.78%)
pelB	PA14_24490	Beta-barrel assembly-enhancing protease	178/494 (36.03%)
pslD	N/A	Hypothetical protein	462/494 (93.52%)
